# Effect of pH and hydroxyapatite-like layer formation on the antibacterial properties of borophosphate bioactive glass incorporated poly(methyl methacrylate) bone cement

**DOI:** 10.3389/fbioe.2024.1462795

**Published:** 2024-09-18

**Authors:** Kara A. Hageman, Rebekah L. Blatt, William A. Kuenne, Richard K. Brow, Terence E. McIff

**Affiliations:** ^1^ Bioengineering Graduate Program, University of Kansas, Lawrence, KS, United States; ^2^ Department of Orthopedic Surgery and Sports Medicine, University of Kansas Medical Center, Kansas City, KS, United States; ^3^ Department of Materials Science and Engineering, Missouri University of Science and Technology, Rolla, MO, United States

**Keywords:** bioactive glass, antibacterial, hydroxyapatite-like layer, periprosthetic joint infection, bone cement, orthopedics

## Abstract

Infection is a leading cause of total joint arthroplasty failure. Current preventative measures incorporate antibiotics into the poly (methyl methacrylate) (PMMA) bone cement that anchors the implant into the natural bone. With bacterial resistance to antibiotics on the rise, the development of alternative antibacterial materials is crucial to mitigate infection. Borate bioactive glass, 13–93-B3, has been studied previously for use in orthopedic applications due to its ability to be incorporated into bone cements and other scaffolds, convert into hydroxyapatite (HA)-like layer, and enhance the osseointegration and antibacterial properties of the material. The purpose of this study is to better understand how glass composition and change in surrounding pH effects the composite’s antibacterial characteristics by comparing the incorporation of 30% wt/wt 13–93-B3 glass and pH neutral borophosphate bioactive glass into PMMA bone cement. We also aim to elucidate how HA-like layer formation on the cement’s surface may affect bacterial adhesion. These studies showed that 13–93-B3 incorporated cements had significant reduction of bacterial growth surrounding the composite beyond 24 h of exposure when compared to a neutral borate bioactive glass incorporated cement (*p* < 0.01) and cement only (*p* < 0.0001). Additionally, through soaking cement composites in simulated body fluid and then exposing them to a bioluminescent strand of *staphylococcus aureus,* we found that the presence of a HA-like layer on the 13–93-B3 or pH neutral glass incorporated cement disks resulted in an increase in bacterial attachment on the composite cement’s surface, where *p* < 0.001, and *p* < 0.05 respectively. Overall, our studies demonstrated that borate bioactive glass incorporated PMMA bone cement has innate antimicrobial properties that make it a promising material to prevent infection in total joint arthroplasties.

## 1 Introduction

Periprosthetic joint infection is the number one cause of failure in total joint arthroplasties within the first 2 years after surgery (Khan et al., 2016). The current rate of periprosthetic joint infection (PJI) in total knee arthroplasty is 0.5%–2% (Ayoade et al., 2021) and it is predicted that by the year 2040, there will be more than 1.4 million total hip arthroplasties and more than 3.4 million total knee arthroplasties. This is a 129% increase in hip arthroplasties and 401% increase in knee arthroplasties when compared to the amount of surgeries in 2014 (Singh et al., 2019). Given the predicted continual increase in volume of surgeries, finding better ways to reduce infection becomes even more pressing.

PMMA bone cement is frequently used in conjunction with total joint arthroplasty for implant anchoring and as antibiotic-loaded cement spacers for use in the treatment of PJI (Jaeblon, 2010). However, PMMA bone cements and spacers offer prime surfaces for bacterial attachment and biofilm formation (Nandakumar et al., 2013; Chang and Merritt, 1992). Biofilm formation has been implicated as a primary contributor to the bacterial resistance and increased difficulty in treating many orthopedic-related infections (Inzana et al., 2016; Ma et al., 2018a; Mandell et al., 2019; Zimmerli and Sendi, 2017). Furthermore, bacterial biofilms are encountered in nearly 65% of postoperative infection cases (Gbejuade et al., 2015), with one of the most common bacteria being *staphylococcus aureus* (Rosteius et al., 2018; Zardi and Franceschi, 2020; Ricciardi et al., 2018). Given the hurdles in treating biofilm-associated infections, it is crucial to pursue ways to better prevent PJI.

The standard preventive measure for PJI is antibiotic incorporation into bone cement which delivers drugs to a targeted location of need, avoiding systemic toxicity (Martínez-Moreno et al., 2017). However, the downsides of this method include poor delivery kinetics that can result in failure of bacteria eradication or even development of antibiotic-resistant bacterial strands (Meeker et al., 2019). Certain antibiotics can also compromise the mechanical properties of the cement (Funk et al., 2019). There is significant debate whether antibiotic loaded bone cement is an effective and efficient method as it lacks solid evidence supporting its prophylactic use clinically (Schwarz et al., 2021; Shahpari et al., 2020). A need remains for materials and techniques that more effectively prevent bacterial attachment and biofilm development.

Bioactive glass is an inorganic material that is commercially used for wound healing as it has the ability to promote angiogenesis and is innately antimicrobial (Ege et al., 2022). In addition to soft tissue applications, bioactive glasses have been shown to enhance bone regeneration as it converts to a hydroxyapatite-like material, which is recognized by and can be integrated into our natural bone tissue (Hench, 1991). Different glass compositions yield different rates of HA-like formation. Borate bioactive glass specifically has been shown to convert into a HA-like layer more rapidly than silicate-based glass (Huang et al., 2006; Rahmati and Mozafari, 2019). While it is known that these glasses have antimicrobial properties, there has been limited investigation into the mechanism of antimicrobial activity. It is speculated to be either from a change in pH in local environment, an increase in osmotic pressure, or due to physical damage to the cell wall (Jung et al., 2019). Given the bioactive glass’s ability to form an HA-like layer and deter bacteria, it is a promising ceramic material that could be incorporated into bone cement for use in total joint replacements.

Previous studies have incorporated borate bioactive glass into bone cement and characterized its HA-like layer conversion and mechanical properties (Funk et al., 2018; Cole et al., 2020a; Cole et al., 2020b; Schumacher et al., 2017).

Other studies have investigated doped bioactive glasses antibacterial activity (Wekwejt et al., 2021; Schuhladen et al., 2020; Miola et al., 2018; Miola et al., 2015), but the innate antibacterial nature of borate-based glass incorporated cement is still understudied, specifically, the bacterial adhesion on the cement surface itself. Additionally, a recently developed pH neutral borate bioactive glass has been formulated to avoid potential negative effects of pH changes in soft tissue applications (Bromet et al., 2023; Freudenberger et al., 2023), but its antimicrobial effects remain unknown. The aim of this study is to (1) further understand the role pH plays in the antibacterial properties of this glass by comparing the effects of 13-93-B3 glass and a pH neutral borophosphate glass and (2) determine the effect an HA-like layer on the surface of bone cement has on bacterial adhesion to the cement.

## 2 Methods and materials

### 2.1 Fabrication of bioactive glass

Bioactive glass was fabricated at Missouri University of Science and Technology (Rolla, MO, United States). Two formulations of glass were made: alkaline borate bioactive glass (13-93-B3) and pH-neutral borophosphate bioactive glass. The glasses nominal molar compositions are given in Table 1. The specific process of glass fabrication for the borate bioactive glass is detailed in other papers (Cole et al., 2020a; Cole et al., 2020b; Funk et al., 2018). The neutral borophosphate glass was made in a similar manner to the 13-93-B3 borate glass. The appropriate raw materials to produce the compositions listed in Table 1 were weighed and combined into a platinum crucible and melted at 1,000°C–1,150°C for 1 h. The melts were stirred with a platinum rod after 30 min and later quenched on steel plates. Glass was milled and passed through a sieve to obtain glass particles <20 µm in size. The particles were stored in a desiccator until combining with the cement.

**TABLE 1:** Bioactive glass compositions (mol%) that were incorporated into bone cement.

	13-93-B3 (B-BG)	pH-neutral (N-BG)
Na_2_O	6	16
K_2_O	7.9	-
MgO	7.7	-
CaO	22.1	24
B_2_O_3_	54.6	40
P_2_O_5_	1.7	20

### 2.2 Cement composite preparation

Commercially available, medium viscosity orthopedic bone cement was combined with the two types of bororphosphate glasses described above. The cement components for both the powder and liquid parts are listed in Supplementary Table S1. A 30% wt/wt glass to dry cement composition was chosen due to previous literature reporting this amount allowed for more HA-like layer formation compared to a 20% wt/wt and also did not compromise the mechanical strength of the cement (Cole et al., 2020a; Cole et al., 2020b). This composite was created by mixing 20 g of DePuy Smartset MV (DePuy, Blackpool, United Kingdom) bone cement powder with 8.57 g of bioactive glass and then mixed with 10 mL of the monomer liquid in a bowl, under vacuum, for 1.5–2 min. The cement was then placed in PTFE molds to form disks, 4 mm thick by 10 mm in diameter, and left in an incubator at 37°C for 1 h to allow for complete curing. Disks were then removed from the molds and disks with major visible defects were discarded. All disks used in experimentation were sterilized in a UV chamber for 5 min prior to submersion in simulated body fluid (SBF) or a bacterial solution. Disks were made with incorporation of basic borate bioactive glass, 13-93-B3, (B-BG) or pH neutral borophosphate bioactive glass (N-BG). Control disks (PCON), that did not contain any glass were created in the same manner. Characterization of the initial B-BG and N-BG composites, prior to submersion, were imaged via SEM/EDS as described below in section 2.4.

### 2.3 Planktonic bacterial viability

For each cement group, B-BG, N-BG, and PCON, 10 cement disks were placed in individual wells of a 24 well plate. A bioluminescent, *staphylococcus aureus* bacterial strain, Xen 36 (ATCC 49525) (Perkin Elmer, Waltham, MA, United States), was then inoculated in Muellar Hinton Broth (MHB) at 10^4^ CFU. 1 mL of this inoculation was placed around each disk. 1 mL of the bacteria only culture as well as 1 mL of MHB only was placed in separate wells and used as controls. After 24 h, the bacteria around each disk and in control wells was plated in triplicate in a black 96 well plate (200 µL/well). The luminescence and absorbance of the plate was read via Synergy H1 Multi-Mode Microplate Reader (Biotek, Winooski, VT, United States). Fresh inoculum at 10^4^ CFU was then placed around the disks again and process was repeated every 24 h for up to 96 h.

### 2.4 Hydroxyapatite formation and characterization

Cement disks were individually placed into 1 mL of simulated body fluid (SBF) (BZ173, Biochemazone, Leduc, Alberta, Canada) to soak in a 24 well plate with their top surface fully submerged in order to pre-form a HA-like layer on the glass incorporated disks. The composition of SBF is located in Supplementary Tables S2, S3. The flat side of the disks that were against the bottom of the PTFE mold during curing were placed facing upwards and used for evaluation. This made for a smoother and more uniform test specimen. Disks were left in the incubator at 37°C for 8 days. Every other day, the entire 1 mL of SBF was removed and replenished with fresh SBF. After day 8, the disks were removed and rinsed in distilled water then set to dry for 24–48 h and stored in a 24-well plate at room temperature.

The flat, top side of both the soaked and non-soaked disks from B-BG, N-BG, and PCON were sputter coated with 5 nm of gold and visualized using Hitachi S4700 Cold-Field Emission Scanning Electron Microscope (SEM) (Hitachi, Tokyo, Japan). The composition of the surface was also found via the X-Max^N^ energy-dispersive X-ray spectroscopy (Oxford Instruments, Abington, United Kingdom). The images of the HA-like layer were taken approximately 3–4 months after the soaking of the disks in SBF was completed.

Additionally, the pH of the SBF surrounding the disks was determined using a Fisher Scientific accumet™ AB15 Basic (Thermo Fisher Scientific, Waltham, MA, United States) pH meter for disks that were soaked for 7 days. SBF was removed and pH was determined on Day 2, 4, 6 and 7 for 10 disks in B-BG, N-BG and PCON.

### 2.5 Bacterial adhesion

**FIGURE 1:**
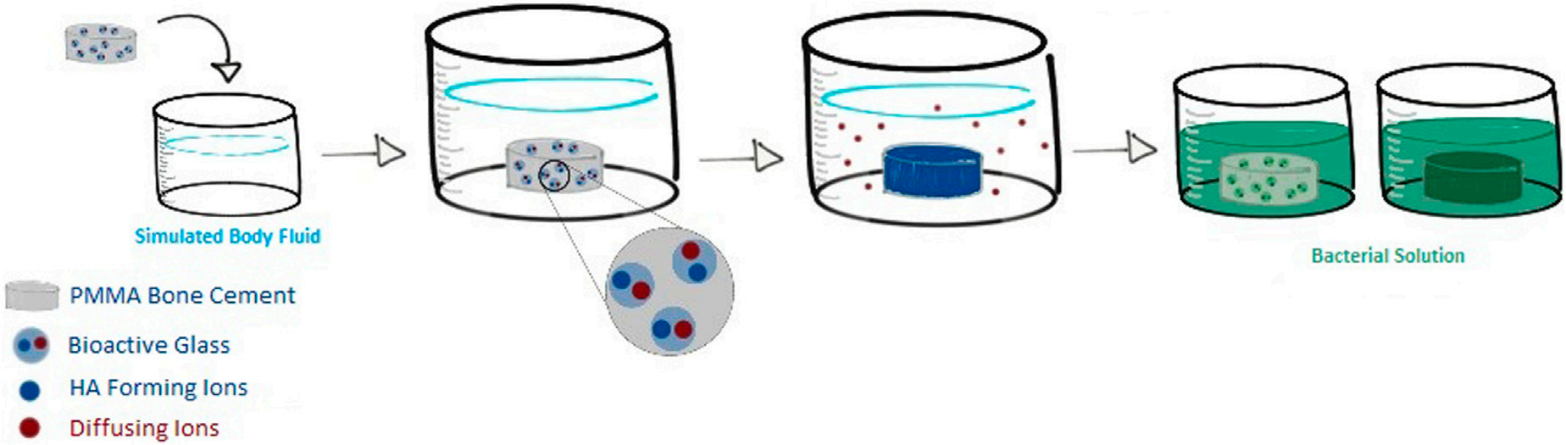
Overview of cement composite testing against bacteria. Glass was incorporated into the cement and formed into disks. Disks were submerged into SBF to form an HA-like layer where they were then removed and tested for bacterial adherence and compared to disk counterparts that were not pre-soaked.

Cement disks from B-BG, N-BG, and PCON groups were tested against two different strains of bacteria, the Xen 36 and Xen 29 (ATCC 12600) (Perkin Elmer, Waltham, MA, United States). Disks from each group with no layer preformed was tested against Xen 29 with an n = 10 for each group. Additionally, the 3 cement groups both with and without a layer preformed were tested with Xen 36 as well. Disks were submerged in 1 mL of MHB inoculated with Xen36 at a 10^6^ CFU concentration, with the goal of identifying the presence of bacteria attached to the disk 48 h after inoculation. The overall process is depicted in Figure 1. At 24 h, disks were rinsed in 1 mL of PBS and placed in fresh MHB inoculated with Xen36. After 48 h, the disks were rinsed and luminescence of the bacteria attached to the disks were imaged using an *in vivo* imaging system (IVIS) (Revvity, Waltham, MA, United States). This gave initial qualitative and quantitative measurements of attached bacteria still on the cement. To confirm these measurements, disks were then placed in a 15 mL falcon tube with 1 mL of fresh MHB. They were vortexed for 10 s, sonicated for 15 min, and then vortexed again for 10 s in order to displace the bacteria from the cement. The sonicated solution was then plated in triplicate in a 96-well plate and luminescence of detached bacteria was read again on IVIS.

### 2.6 Statistical analysis

In order to determine statistical difference between B-BG, N-BG, and PCON, both soaked and non-soaked, data was first tested for normality utilizing a Shapiro-Wilks test (GraphPad Prism 9.3.1 software). Once confirmed, a Welch’s t-test was performed comparing the groups to one another for both the adherence and planktonic bacterial viability studies. Significance between groups was presented as 95% confidence intervals.

## 3 Results

### 3.1 Composite characterization

**FIGURE 2:**
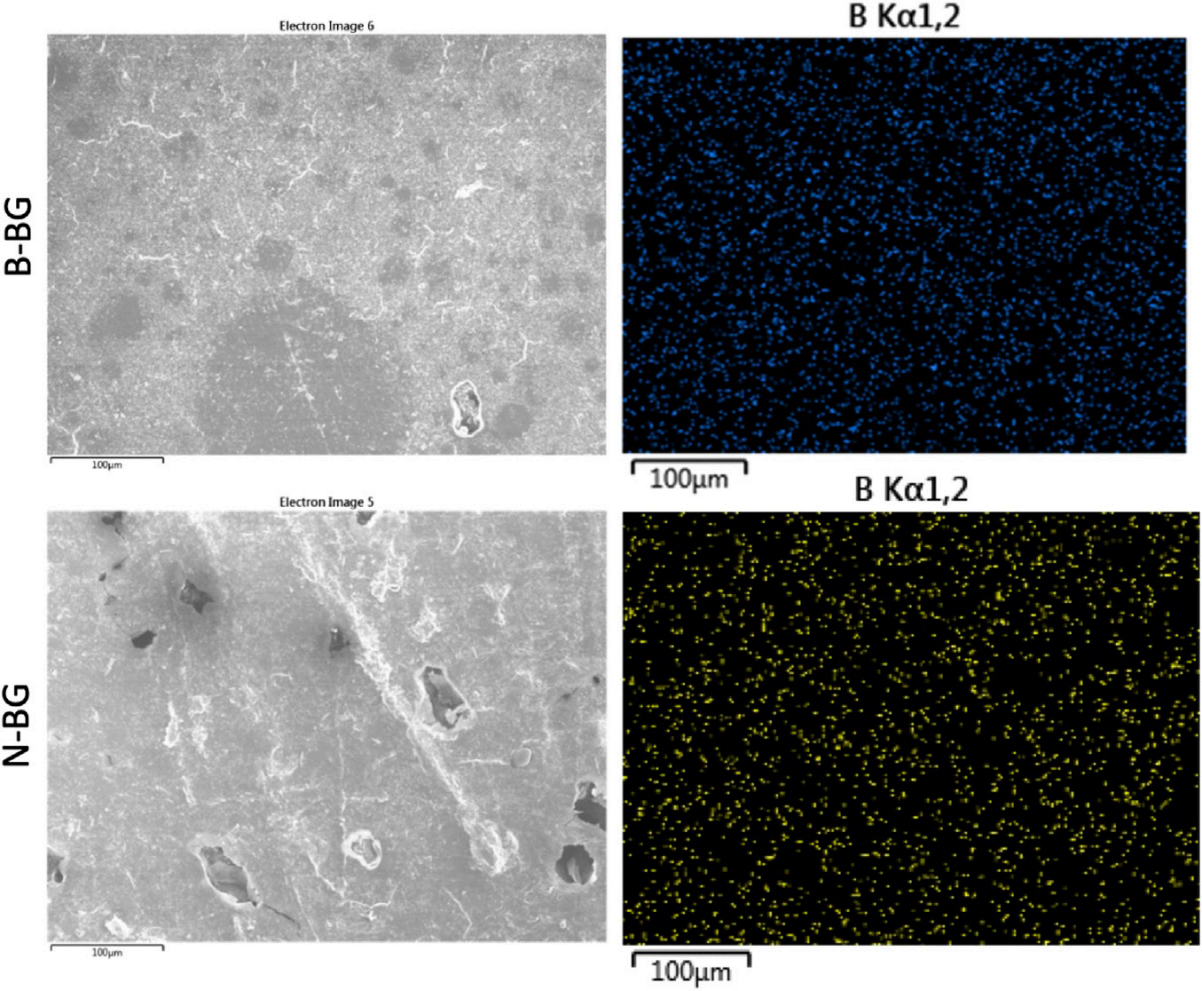
SEM and EDS mapping of boron in the 13-93-B3 incorporated cement (top) and the borophosphate pH neutral glass incorporated cement (bottom). These images were taken prior to soaking in SBF or bacterial inoculum.

The B-BG and N-BG composites were characterized via SEM/EDS prior to submersion in SBF or a bacterial inoculum. Figure 2 displays EDS mapping of boron evenly dispersed on the composite’s surface in both the B-BG and N-BG composite prior to submersion in SBF. Table 1 displays the composition of the two different glasses, and the mappings of the different elements in each composite are shown in Supplementary Figure S1.

### 3.2 Alkalinity

**FIGURE 3:**
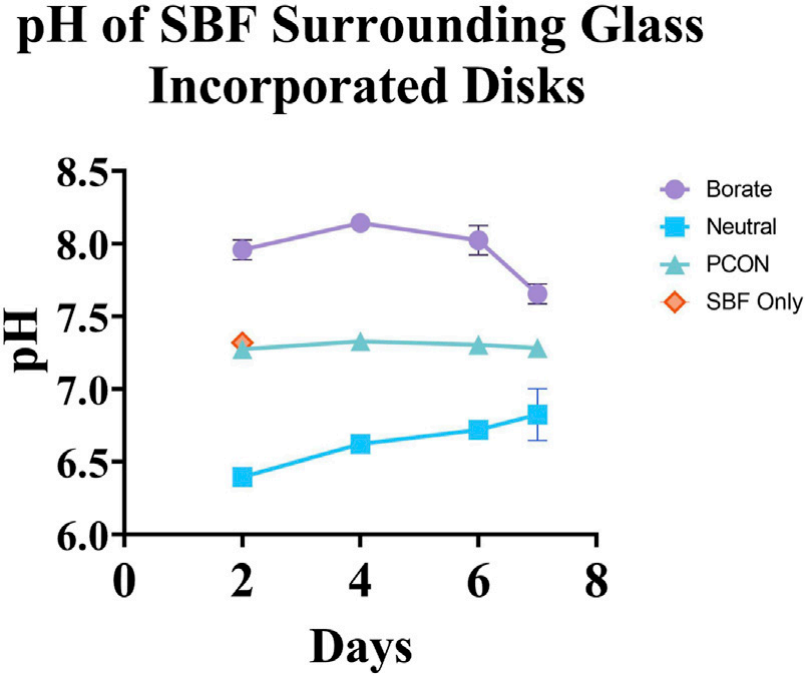
pH of the simulated body fluid surrounding each cement composite over the course of 7 days. The error bars represent a 95% CI and there was a n = 10 for each group point plotted.

The pH of SBF surrounding B-BG and N-BG is depicted in Figure 3. Disks with no glass incorporated (PCON) and SBF only were used as controls. There was no significant difference between PCON and SBF only at Day 2 and therefore PCON was used as main control for the remainder of the days. SBF surrounding the PCON disks were shown to have a consistent pH value throughout the 7-day period. The SBF surrounding B-BG disks showed a more basic pH with a slight drop in pH from day 6–7. The pH was above 7.5 for all 8 days. The SBF surrounding N-BG disks conversely showed a more acidic pH which rose slightly from day 6–7. The average pH was below 7.0 for the 7 days.

### 3.3 Planktonic bacterial viability surrounding the disks

**FIGURE 4:**
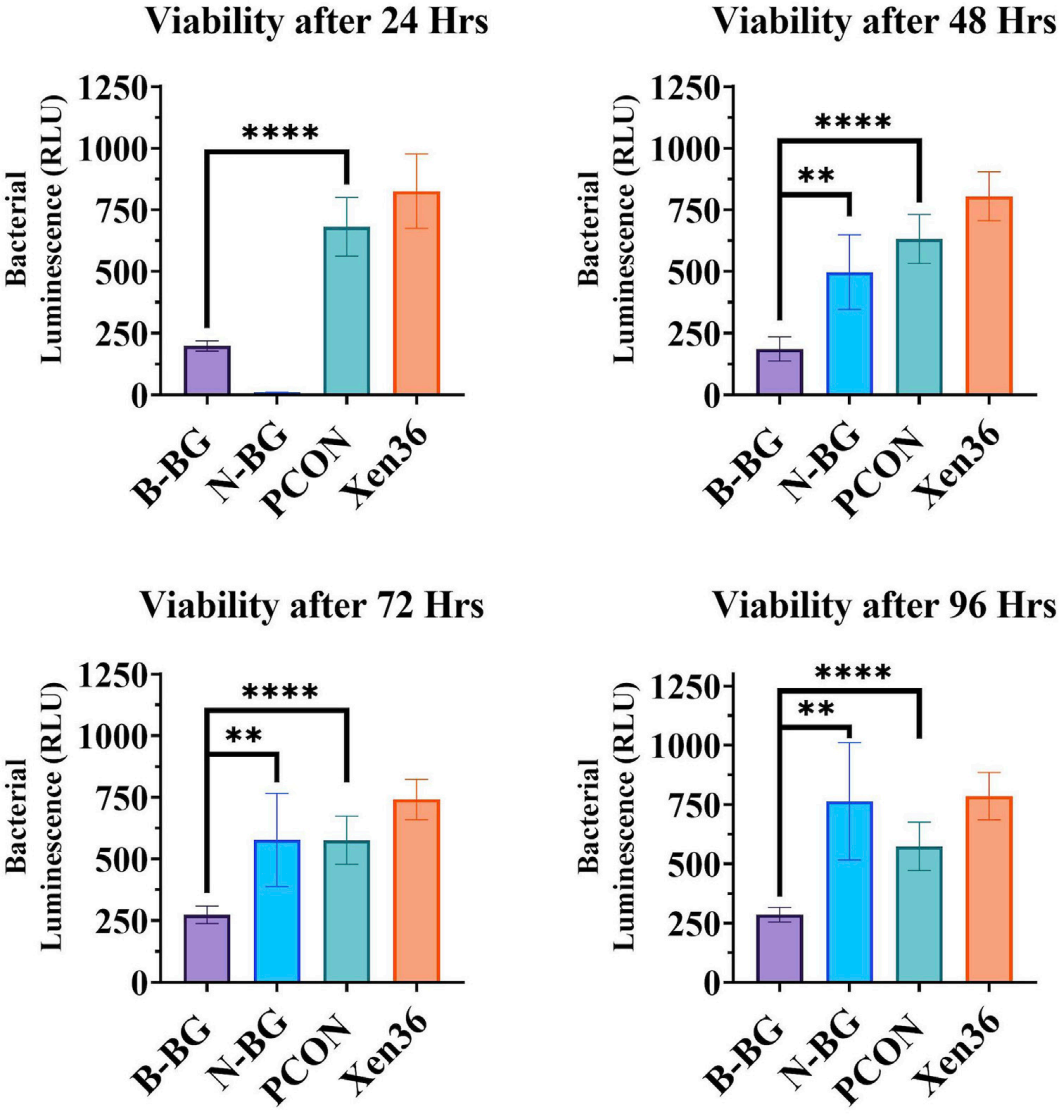
Comparison of the bacterial luminescence in the MHB surrounding the 13-93-B3 bioactive glass incorporated cement, pH neutral glass incorporated cement, cement only. Bacteria only is also displayed as a control. Graph in the top left represents bacteria luminescence after 24 h of the disks being submerged. Moving clockwise, 48 h, 96 h, and 72 h are displayed. Error bars on all graphs represent a 95% CI with an n = 10 for each group. **p < 0.01, ****p < 0.0001.

When testing the viability of the planktonic bacteria surrounding the different disks, we found a significantly lower bacterial luminescence in the sample from the B-BG disks compared to PCON from all timepoints (24–96 h) (*p* < 0.0001 at each timepoint). B-BG also had significantly less bacterial luminescence when compared to the N-BG disks at the 48 (*p* = 0.0010), 72 (*p* = 0.0054), and 96 (*p* = 0.0017) hour timepoints as seen in Figure 4. There was also significantly less luminescence from the bacteria surrounding the N-BG disks compared to PCON (*p* < 0.0001) at 24 h. However, for 48–96 h there was no significant difference noted between the bacteria surrounding the N-BG disks and that surrounding the PCON disks.

### 3.4 HA-like layer formation

**FIGURE 5:**
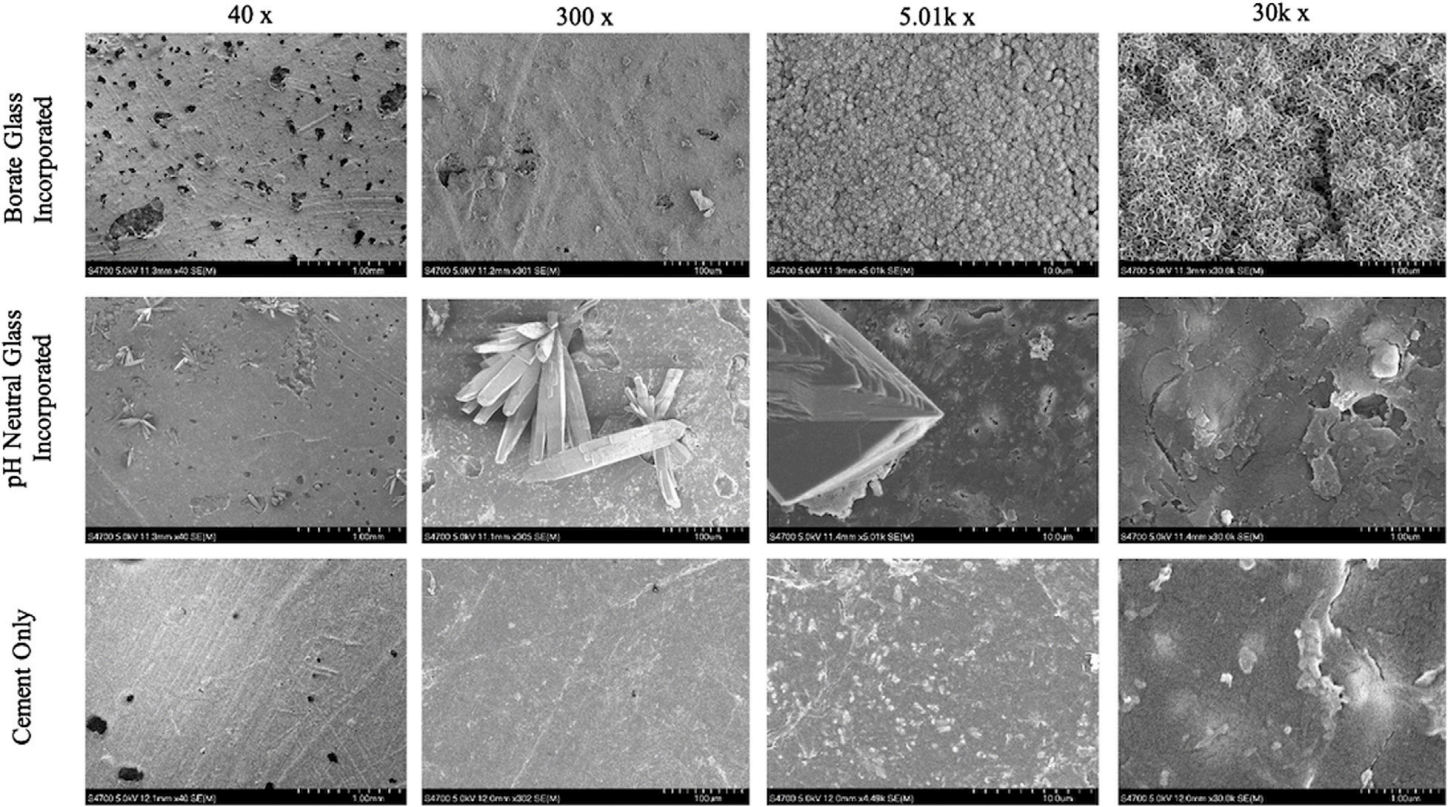
SEM images of glass incorporated disks with disks soaked for 8 days in SBF on the bottom row. 13-93-B3 bioactive glass cement disks are on the top, pH neutral glass incorporated disks in the middle and cement only pictured on the bottom. All images were taken at 40x, 300x, 5.01k x, and 30k x respectively moving left to right.

**FIGURE 6:**
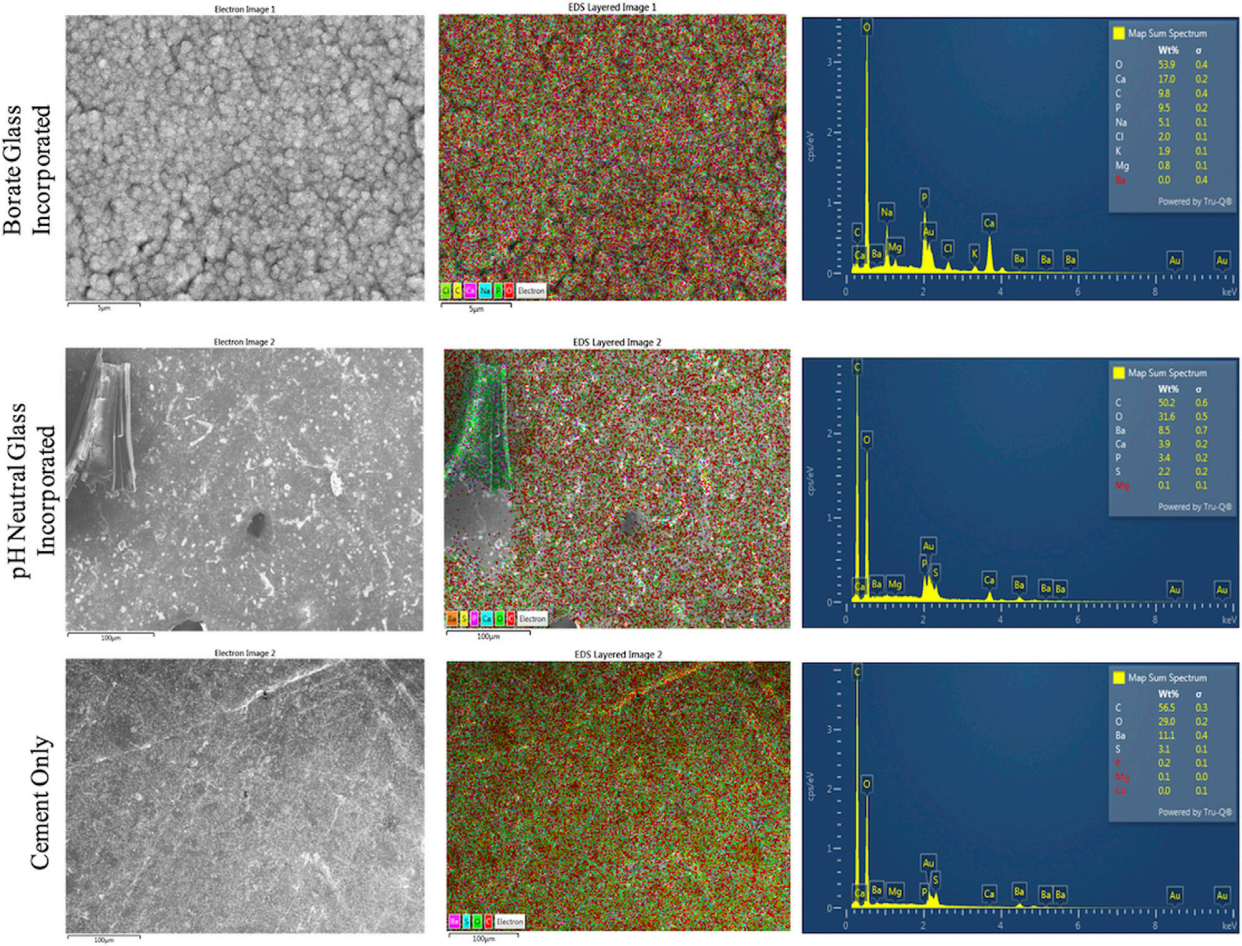
EDS analysis of disks soaked in SBF for 8 days. 13-93-B3 bioactive glass incorporated disks are on top, pH neutral glass incorporated disks in the middle, and cement only is on the bottom. The different elements present on the surface are shown as an overlay SEM image in the center, and the elements are graphed and presented in a table on the images to the right.

SEM/EDS images of the cement groups after soaking in SBF for 8 days are shown in Figures 4, 5. The B-BG disks showed HA-like formation at the magnification of 5.01k and 30k. From EDS in Figure 6, B-BG showed a consistent calcium phosphate presence across the surface as well as magnesium. The Ca:P ratio on the B-BG was seen to be ∼1.7. The N-BG cement also shows hydroxyapatite-like surface, with the morphology of the calcium phosphate taking form as a brushite crystal. These were much larger crystals and were viewable at a magnification of 40 and 300 as seen in Figure 5. The bulk of the crystal appeared on EDS as calcium phosphate, whereas the remaining area of the surface showed as barium, sulfur, oxygen, and carbon. The Ca:P ratio on the N-BG was seen to be ∼1.1. The scan of the PCON showed a flat surface with just carbon, oxygen, barium, and sulfur. The barium and sulfur elements are present due to barium sulfate being a component of the commercial PMMA bone cement (Supplementary Table S1) in order to provide radiopacity.

### 3.5 Bacterial adherence on disk surface

**FIGURE 7:**
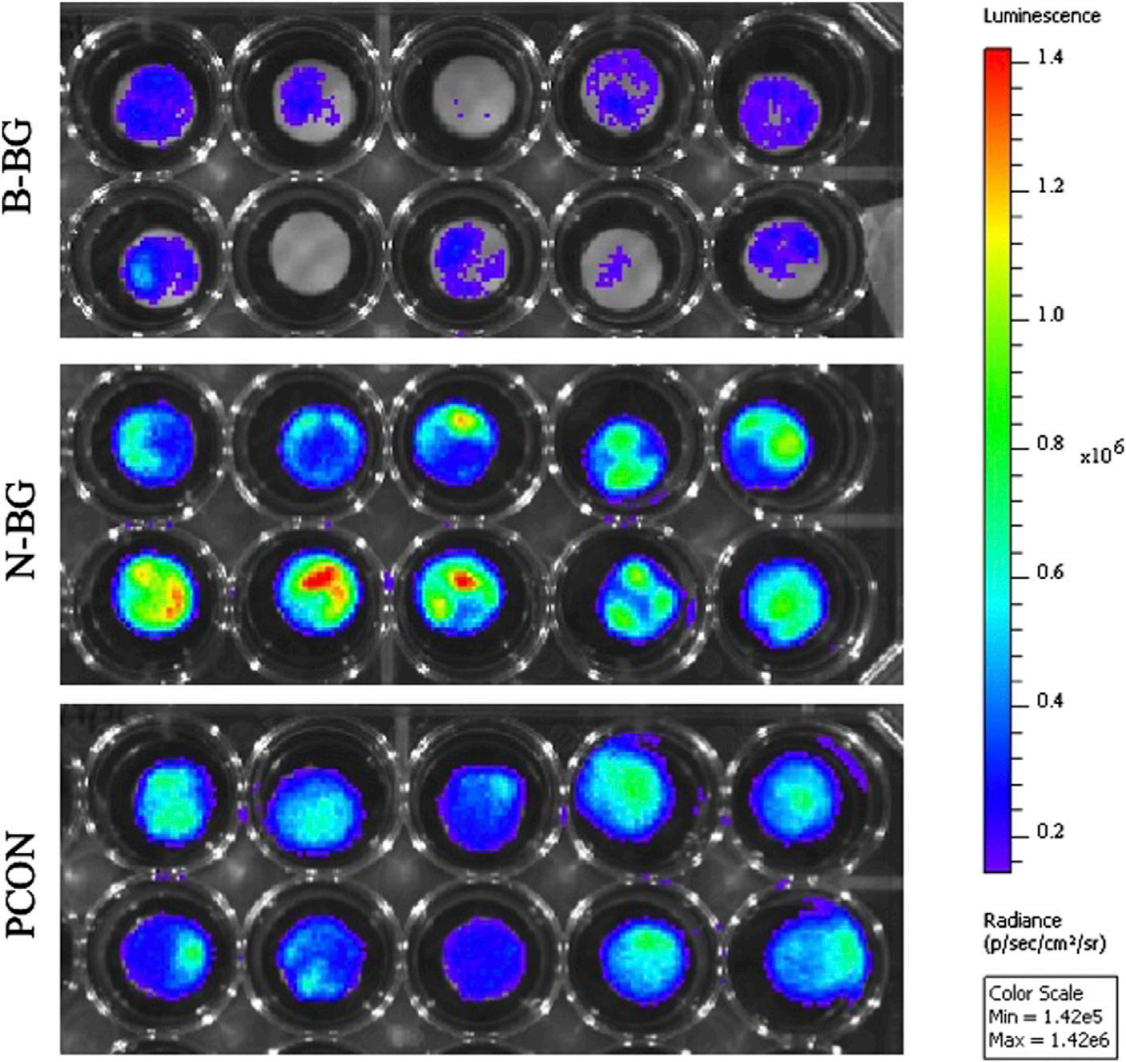
IVIS image after 48 h of Xen 29 bacterial exposure. B-BG disks are on the top, N-BG in the middle and PCON on the bottom.

**FIGURE 8:**
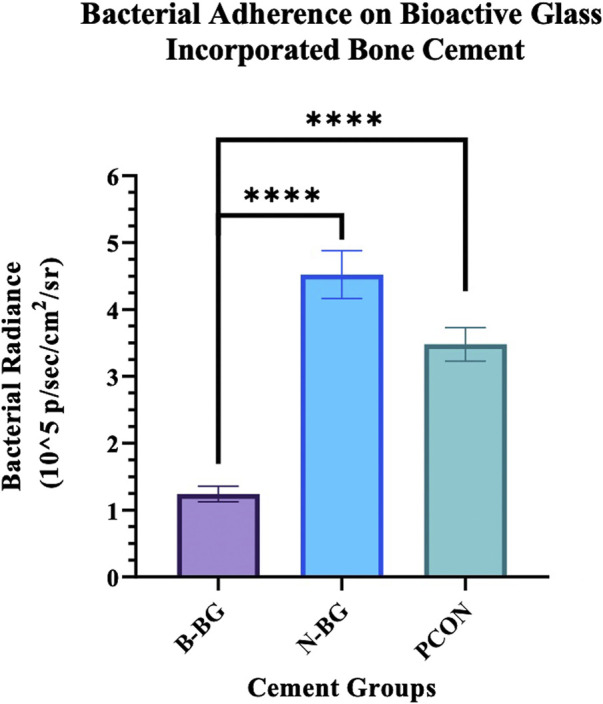
Quantification of the Xen 29 bacterial luminescence on the different cement groups after 48 h of bacterial exposure. Error bars represent 95% confidence interval and n = 10 for each group. ****p < 0.0001.

The difference in Xen 29 attachment between the cement groups after 48 h of incubation is shown in Figures 7, 8. In Figure 7 the decrease in bacterial adherence on the B-BG disks is qualitatively shown as there is weaker luminescence and it is not fully spread across the whole of the disks as compared to the N-BG or PCON disks. Figure 8 quantitatively shows the significant reduction of bacterial luminescence from the B-BG composites when compared to the N-BG (*p* < 0.0001) and PCON (*p* < 0.0001).

**FIGURE 9:**
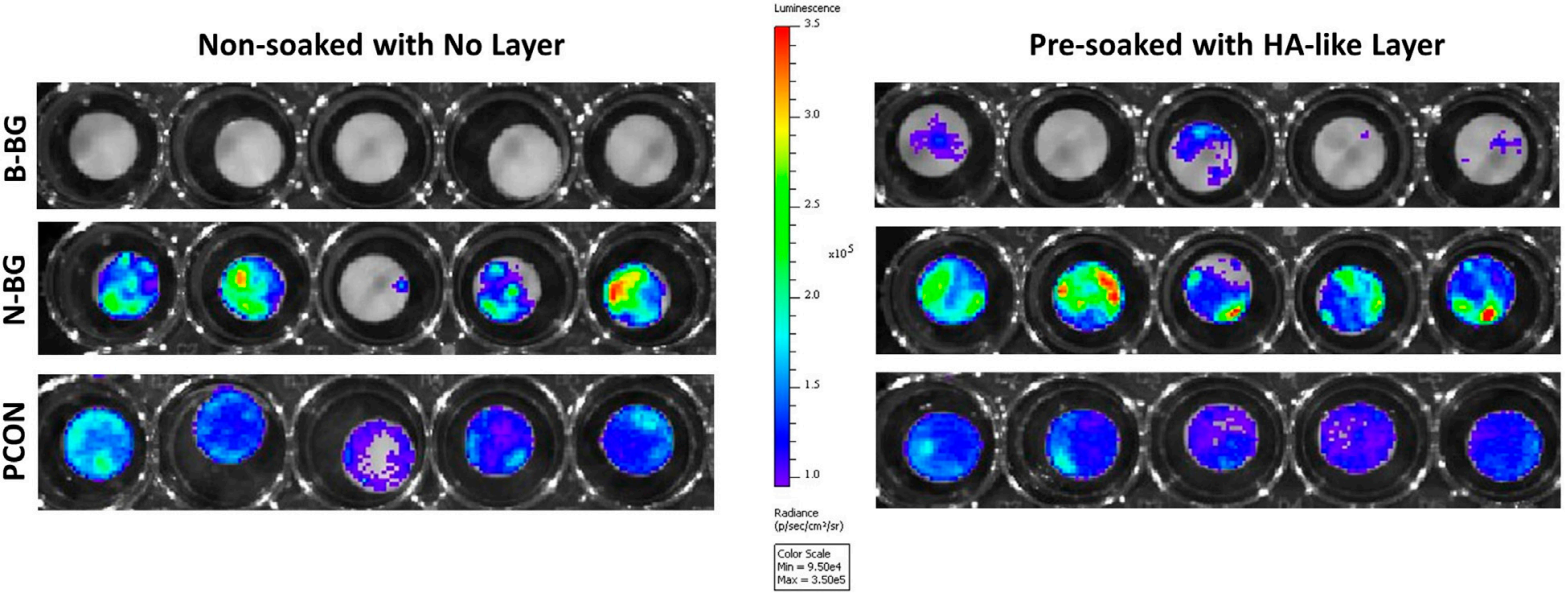
Image from IVIS that demonstrates bacterial luminescence for bacteria adhered on the surface of the cement disk groups after 48 h. 13-93-B3 glass disks are on the top, pH neutral in the middle and PCON on the bottom. Those non-soaked are on the left and those soaked for 8 days in SBF prior to bacterial exposure are represented on the right. Scale is adjusted to represent luminescence relative to the borate bioactive glass, non-soaked, disks.

**FIGURE 10:**
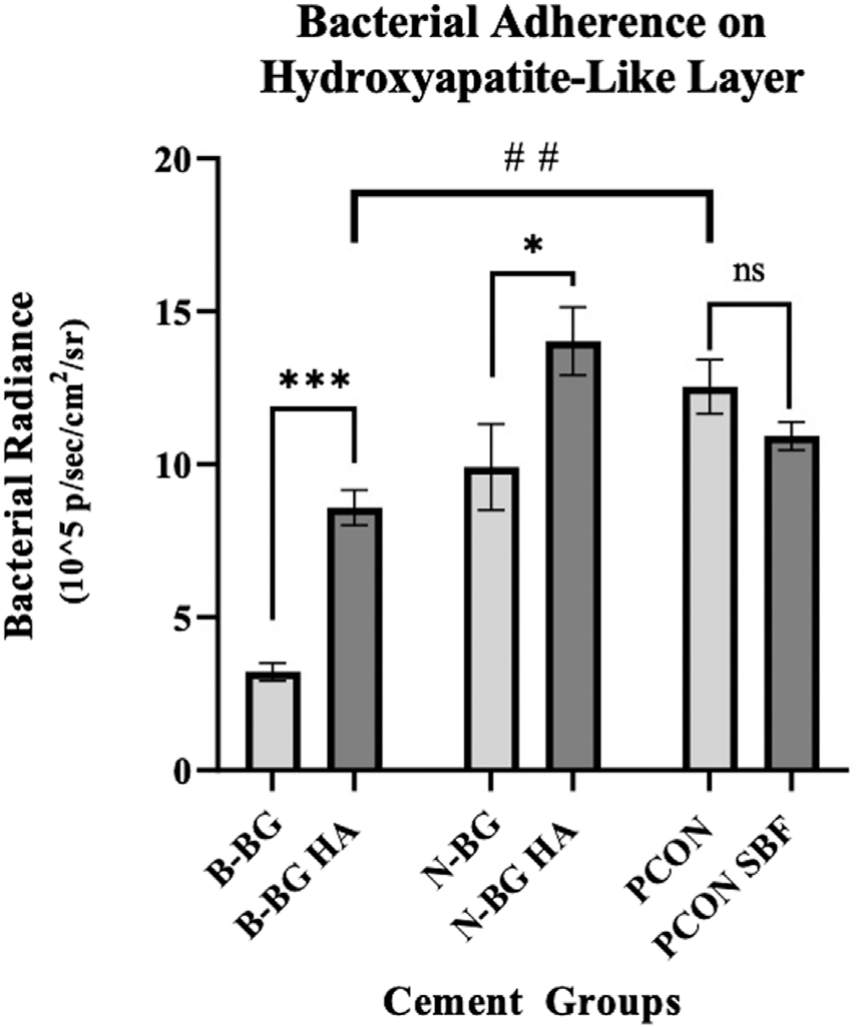
This graph compares bacterial adherence on cement disks that were pre-soaked in SBF to those that were not soaked, after 48 h of bacterial incubation. Error bars represent standard error with each group having an n = 10. *p < 0.05, **p < 0.01, ***p < 0.001, ns not significant.

The Xen 36 attachment both on disks with and without a preformed layer are shown in Figures 9, 10. Figure 9 qualitatively shows the bacteria coverage on all cement groups relative to B-BG non-soaked group. It is evident that the B-BG and N-BG samples that were soaked prior to form an HA-like layer, have higher luminescence compared to their non-soaked counterparts. No significant difference is seen between PCON groups, soaked or non-soaked.

Quantitatively, Figure 10 shows that after 48 h of bacterial incubation there was significantly less bacterial attachment to B-BG disks compared to N-BG and PCON disks with *p* = 0.001 and *p* < 0.0001 respectively. Pre-forming an HA-like layer by soaking a disk in SBF prior to bacterial incubation had a significant effect on bacterial adherence. Figure 10 shows that disks with a pre-formed HA-like layer had significantly higher bacterial luminescence indicating more bacterial adherence. When comparing B-BG disks with an HA-like layer to B-BG disks with no pre-formed layer, those with the layer had more attached bacteria (*p* < 0.0001). This same trend was seen when comparing N-BG disks with an HA-like layer and N-BG disks with no pre-formed layer with a *p*-value = 0.035. No significant difference was seen between the PCON samples that were pre-soaked in SBF and those not soaked. Finally, Figure 10 also demonstrates that while the B-BG disks with a pre-formed HA-like layer had more bacterial attachment than B-BG disk alone, this is still significantly less than the bacteria attached to PCON samples with a *p*-value = 0.0018.

The data collected after vortexing and sonicating the cement disks were similar to the radiance values obtained from disk’s surfaces (prior to sonification) and can be seen in Supplementary Figures S2, S3.

## 4 Discussion

With total joint arthroplasty procedures predicted to rise, and bacteria developing resistance to antibiotics, infection is becoming an ever-pressing issue for the orthopedic community. Previous work has shown bioactive glass incorporated cement as a promising material in orthopedics due to its mechanical stability and its bioactive nature that allows for better osseointegration and enhanced biocompatibility (Bistolfi et al., 2019). Cole et al. (2018), tested 13–93-B3 glass incorporated cement with up to 40% wt/wt loading that maintained mechanical strength above the ASTM F451-16 threshold of 70 MPa for weight bearing cement. Additionally, Cui et al., found bioactive glass loaded PMMA cement from 10 to 30 wt/wt% maintained the necessary mechanical properties and further showed bioactivity ability and biocompatibility *in vivo*. They found that 30% glass loaded PMMA cement had significant osteoinduction and increased the amount of bone formation surrounding the interface of the implant when compared to PMMA cement alone (Cui et al., 2017).

In terms of the antibacterial properties of bioactive glass incorporated cement, studies have demonstrated the ability for a combination of bioactive glass and antibiotic loaded bone cement to extend the elution of antibiotic from the cement (Funk et al., 2018). Others have also looked at preliminary antibacterial studies with incorporating doped silicate-based glasses into PMMA cement and have shown significant reduction in *S. epidermidis* biofilm development as well as inhibitory effect against *Staphylococcus aureus*, *Escherichia coli*, *Bacillus* and *C. albicans* (Miola et al., 2018; Verné et al., 2019). While promising for infection prevention, there remains a need to better understand the innate antimicrobial properties of borate bioactive glass, as well as to understand how the bioactivity and formation of an HA-like layer might alter the susceptibility of cement to bacterial adhesion. Methods that allow for visualization of bacteria adhering onto the implant also add to the strength of *in vitro* antimicrobial testing. Better understanding the antimicrobial properties of a glass incorporated cement composite will help determine if borate bioactive glass incorporated cement can be utilized as a prophylactic measure for periprosthetic joint infection.

The antimicrobial properties of bioactive glasses have mainly been attributed to changes in pH and osmolarity in the surrounding environment (Jung et al., 2019; Drago et al., 2018; Coraça-Huber et al., 2014; Hoppe et al., 2011). This environment induces stress on bacterial cells, including the orthopedic relevant strand, *S. aureus* (Rosteius et al., 2018; Ma et al., 2018a). Most previous studies have looked at the properties and effects of silicate based bioactive glasses, (Drago et al., 2014; Zhang et al., 2010; Miola et al., 2014), however, the higher percentage of borate in borate based glasses causes them to dissociate faster leading to a more rapid change in pH and a faster rate of formation of a HA-like layer (Fu et al., 2010; Huang et al., 2006).

In order to understand the role of pH effect on bactericidal ability, this study sought to compare the more basic borate bioactive glass, 13–93-B3, to a pH neutral borophosphate bioactive glass. The composition of neutral glass shown in Table 1 is also considered “fast-acting” according to literature (Bromet et al., 2023). By submerging the glass incorporated cement into SBF for 7 days, we saw an altering of pH in both the cement disks with B-BG and N-BG as compared to the PCON. It is known that phosphate-based glasses decrease a solution’s pH, but borate-based glasses increase the pH (Bromet et al., 2023; Goetschius et al., 2018; Ma et al., 2018b). This aligns with the results shown in Figure 3. This study confirmed that the bioactive glasses still cause change in the pH of the local environment even after being loaded into PMMA bone cement at 30% wt/wt.

Furthermore, Figure 4 demonstrates that after the initial 24 h, both B-BG and N-BG disks can significantly decrease the bacterial viability in the microenvironment. While this bacterial viability experiment was performed in an MHB solution and not an SBF solution, it indicates that changes in pH may not be the only factor contributing to the observed antibacterial activity. We suspect that a drastic change in osmotic pressure due to a burst release of ions within the first 24 h decreased bacterial viability. However, beyond 24 h, the viability of the bacteria surrounding the N-BG was not significantly different compared to that of the PCON control samples for up to 96 h as displayed in Figure 4. The bacteria surrounding the B-BG disks however was significantly less than the bacteria surrounding the PCON at all timepoints. The 13–93-B3 glass’s ability to sustain a higher pH for longer duration might be contributing to the observed decrease in bacterial viability surrounding the B-BG at all timepoints. Additionally, the presence of Mg in the 13–93-B3 glass may also contribute to the B-BG composite’s prolonged antibacterial activity when compared to the N-BG composite. It is important to note that while both glass types are fast-reacting, the rate of degradation may not be the same and therefore would induce different osmotic pressures on the bacteria in the surrounding environment within the first 24 h of incubation. Further studies would need to look at determining the concentration of released ions from the B-BG and N-BG glasses that could be contributing to the osmotic pressure on the bacteria and the rates of degradation and conversion of the glass.

One of the reasons bioactive glass demonstrates promise in bone regeneration applications is that as it dissolves it transforms into a HA-like substance, forming a layer on the surface of surrounding materials, that has properties similar to the mineral found in natural bone and teeth (Hench, 1991). Incorporating borate bioactive glass into tissue scaffolds has previously been shown to increase the bioactivity of a material by enhancing osteointegration (Fu et al., 2010; El-Rashidy et al., 2017). Bioactive glass has also previously been incorporated into PMMA bone cement (Cui et al., 2017; Wekwejt et al., 2021). As discussed above, different compositions of glass can alter the rate of this formation but different compositions and production procedures also can lead to different forms of this HA-like layer (Huang et al., 2006) which in turn might affect the biological properties of the materials (Kargozar et al., 2019).

In reference to the layer formation on our B-BG and N-BG disks, it is clear that the morphology of the HA-like layer formed was different. Figure 5 displays crystals on N-BG disks that were visible at a lower magnification and resemble a brushite layer formation on the surface of the cement. This is consistent with previous literature showing differences in X-ray diffraction data for borophosphate glasses reacted in SBF for 8 days (Bromet et al., 2023; Freudenberger et al., 2023) and may be due to the local pH at the glass-solution interface. For example, the Ca:*p* ratio of the amorphous precipitation phase that formed when soluble borate glasses reacted in phosphate-containing solutions systematically increased with increasing solution pH (Shen et al., 2022). Figure 6 demonstrates that the N-BG surface was also not as fully covered in calcium phosphate when compared to the B-BG composite. This difference in layer formation could explain a difference in bacterial adhesion.

While we have shown B-BG and N-BG incorporated cement can decrease the viability of bacteria in the microenvironment surrounding the cement, we see a similar trend when looking at the adhesion of the bacteria to the surface of these disks. Utilizing an additional strain of bioluminescent *S. aureus*, Figures 7, 8 show a significant decrease in bacteria on the surface of the B-BG compared to N-BG or PCON. This could again be attributed to the 13–93-B3 surface pH being higher or potentially the presence of Mg in the glass. A similar trend was seen with the Xen 36 bacteria in Figures 9, 10. These figures display the difference in the number of bacteria found on the surface with B-BG having less bacteria when compared to the PCON and N-BG after being exposed to bacteria for 48 h. However, as discussed above, the surface of this cement changes as the glass converts into a HA-like layer. While this is beneficial for osseointegration, prior literature has shown increasing HA layer content was associated with greater bacterial adherence (Liu et al., 2013). It is well known that the properties of the surface of an implant, including roughness, porosity, surface chemistry, and hydrophobicity, can affect susceptibility for bacterial attachment (Nandakumar et al., 2013). This is why it is important to consider how changes in the surface layer on the cement would affect the attachment. Figure 10 also demonstrates that both B-BG and N-BG disks show an increase in bacterial attachment when a layer is pre-formed on the cement’s surface. However, the B-BG disks with an HA-like layer still have significantly less bacteria attached when compared to the PCON control. The B-BG disks also have less bacterial adherence than the N-BG disks which could be due to difference in HA-like layer morphology and/or difference in pH and surface chemistry. Figure 10 shows the radiance of the attached bacteria on the cement. Supplementary Figure S3 shows the radiance of the bacteria that was attached to the cement after vortexing and sonicating the disks. While sonication is a more accepted method of removing bacteria from an implant, (Karbysheva et al., 2020; Bjerkan et al., 2009), there is concern that sonication would release more glass into the solution and therefore kill more bacteria and skew results. Despite this concern, our data showed the same trends that we display in Figure 10, further validating our findings. Moving forward, more effort should be taken to engineer the composite in a way that will perpetuate bactericidal properties while still allowing time for the bone cells to form a solid attachment to the cement.

This study is limited in that all *in vitro* studies were run under static conditions. Given the dynamic nature of the body and fluid exchange that occurs *in vivo*, it is important to better understand the sustainability of the antibacterial properties of the glass incorporated cement once under dynamic conditions. Also, the use of bioluminescent bacteria for these studies, it is important to note that the strength of luminescence of the bacteria is connected with the bacteria’s metabolic activity. Staining techniques would need to be utilized to fully quantify the number of dead bacteria or determine if biofilm formation is prevented. Finally, the SEM/EDS images of the surface were taken several months after the soaking occurred which could have led us to observing a further developed crystalline structure from moisture in the air than when the disks were first removed from the solution.

Future studies aim to enhance the antibacterial properties of 13-93-B3 glass incorporated cement by doping the glass with metal ions in order to maintain an antibacterial microenvironment for a longer period of time. Furthermore, the antibacterial properties and biocompatibility of the glass incorporated cement composites will be tested using a dynamic system that better represents *in vivo* conditions. While previous literature has reported cytotoxicity of borate bioactive glass when tested *in vitro* due to large concentrations of B and Mg in the cell media (Lopes et al., 2013), *in vivo* studies report borate bioactive glass enhanced osseointegration in bone defect models (Zhang et al., 2015; El-Rashidy et al., 2017; Kunisch et al., 2023; Jung et al., 2012). This is why it will be important to test the biocompatibility in a dynamic environment and also ensure the antibacterial properties are maintained in a dynamic model.

## 5 Conclusion

Given the growing popularity of bioactive glass usage in soft and hard tissue engineering, there is opportunity to better optimize its usage in orthopedic applications. By comparing the incorporation of 13-93-B3 and a pH neutral borophosphate glass into PMMA bone cement, this study furthers the understanding of each type of glass’s antibacterial properties. The pH neutral glass composite shows an initial bactericidal ability, however, the 13-93-B3 incorporated cement demonstrated prolonged antibacterial activity. This is evidenced through a decrease in bacterial viability surrounding the 13–93-B3 cement composite for up to 96 h of bacterial exposure. This indicates that a glass-induced change in surrounding environmental pH and/or presence of Mg plays a role in the prolonged antimicrobial properties the cement composite. This study also demonstrates the bioactivity of the cement composite through its ability to form a hydroxyapatite-like layer and how this layer increases the surface’s susceptibility for bacterial attachment. Overall, this paper displays the potential of incorporating borate bioactive glasses into bone cement to be used in total joint arthroplasties to prevent the development of periprosthetic joint infection.

## Data Availability

The raw data supporting the conclusions of this article will be made available by the authors, without undue reservation.
