# Supplementary material for: Effect of pH and hydroxyapatite-like layer formation on the antibacterial properties of borophosphate bioactive glass incorporated poly(methyl methacrylate) bone cement
Source: Front Bioeng Biotechnol. 2024 Sep 18;12:1462795. doi: 10.3389/fbioe.2024.1462795 (PMC11445002; doi:10.3389/fbioe.2024.1462795)
Supplement: Supplementary file 1 [file DataSheet1.docx]

**Supplemental Materials**

Supplementary Table 1. Components of DePuy SmartSet Medium Viscosity Bone Cement.

Supplementary Table 2. A table obtained from Biochemazone with the ion concentration present in the simulated body fluid and its comparison to blood plasma.

Supplementary Table 3. A table obtained from Biochemazone with the order and amount of reagents combined to make the SBF.

Supplementary Figure 1. EDS mapping of the different elements found in 13-93-B3 glass incorporated cement (A) and of pH neutral borophosphate incorporated cement (B). The barium and sulfur are components of the PMMA cement and the other elements are found in each of the respective glass compositions.

Supplementary Figure 2. Xen 29 bacteria sonicated off of the cement groups after 48 hours of incubation as well as bacteria only and MHB only controls, with an n=10 for each group. Error bars represent standard error with ****p<0.0001.

Supplementary Figure 3. This graph shows the radiance of Xen 36 bacteria that was sonicated off of the different cement disk groups after 48 hours of bacterial incubation. Error bars represent standard error with each group having an n=10. ## p=0.01, **** p<0.0001, ns not significant

| Components of Smartset MV Bone Cement | |
| --- | --- |
| Powder | **Composition (%w/w)** |
| Polymethylmethacrylate | 67.05 |
| Methylmethacrylate / Styrene Copolymer | 21.10 |
| Benzoyl Peroxide | 1.85 |
| Barium Sulphate | 10.00 |
| Liquid |  |
| Methylmethacrylate | 98.00 |
| N,N-Dimethyl-p-toludine | $\leq$2.00 |
| Hydroquinone | 75 ppm |

| Supplemental Table 2. Concentration of Ions in SBF and human blood plasma (mM) | | |
| --- | --- | --- |
| Ion | **Simulated Body Fluid** | **Blood Plasma** |
| Na^+^ | 142.0 | 142.0 |
| K^+^ | 5.0 | 5.0 |
| Mg^2+^ | 1.5 | 1.5 |
| Ca^2+^ | 2.5 | 2.5 |
| Cl^-^ | 148.8 | 103.0 |
| HCO^3-^ | 4.2 | 27.0 |
| HPO_4_^2-^ | 1.0 | 1.0 |
| SO_4_^2-^ | 0.5 | 0.5 |

| Supplemental Table 3. SBF Reagents (pH 7.40, 1L) | | |
| --- | --- | --- |
| Order | **Reagent** | **Amount** |
| 1 | NaCl | 7.996 g |
| 2 | NaHCO_3_ | 0.350 g |
| 3 | KCl | 0.224 g |
| 4 | K_2_HPO_4_•3H_2_O | 0.228 g |
| 5 | MgCl_2_•6H_2_O | 0.305 g |
| 6 | 1M-HCl | 40 mL |
| (About 90% of total amount of HCl to be added) | | |
| 7 | CaCl_2_ | 0.278g |
| 8 | Na_2_SO_4_ | 0.071 g |
| 9 | (CH_2_OH)_3_CNH_2_ | 6.057 g |

Supplemental Figure 1.


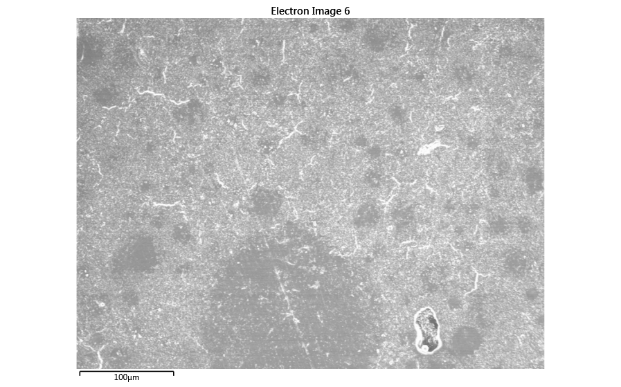

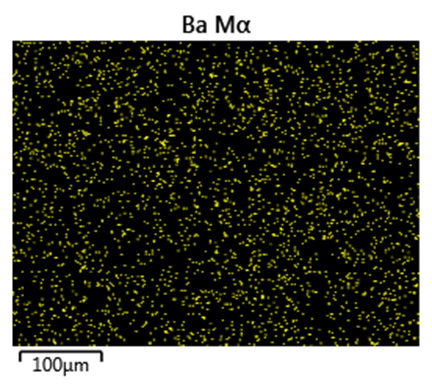

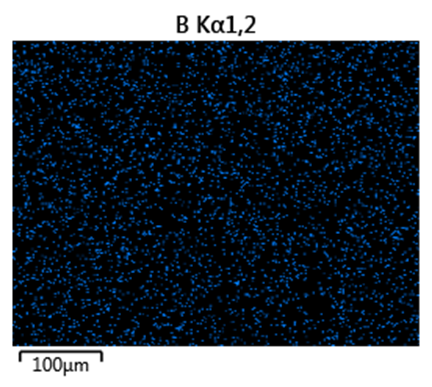

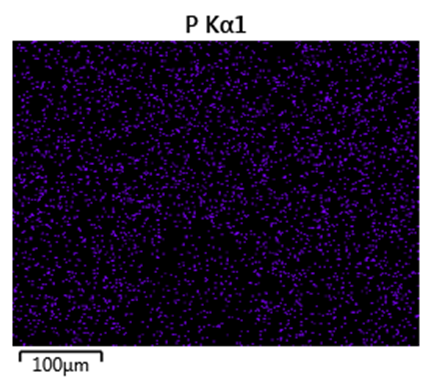

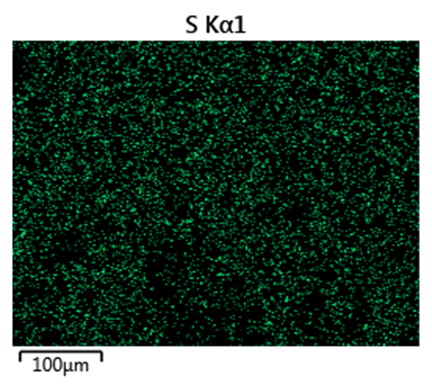

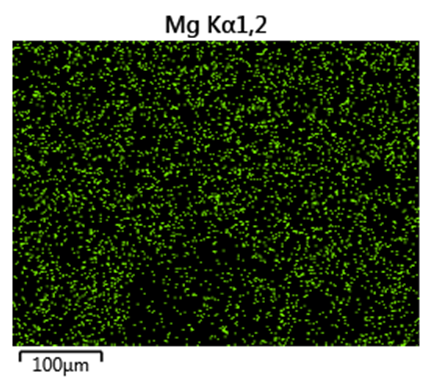

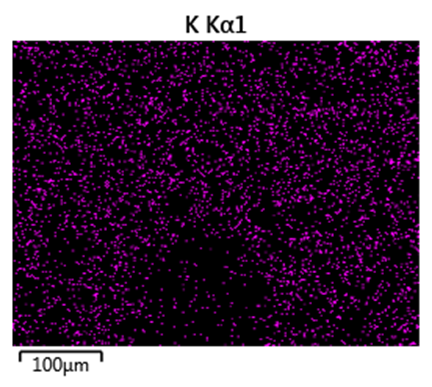

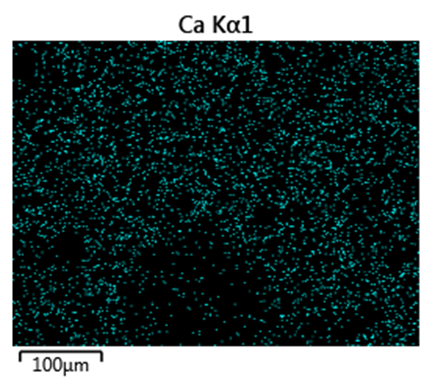

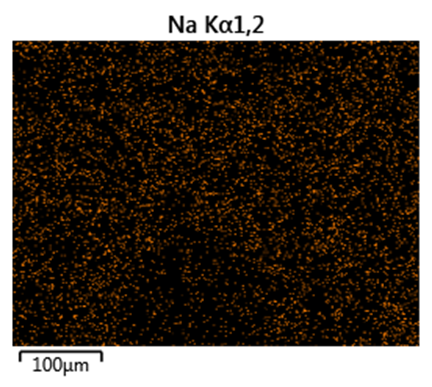


A)


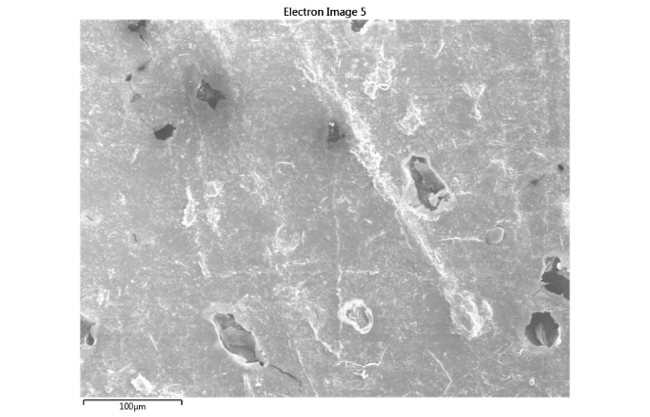

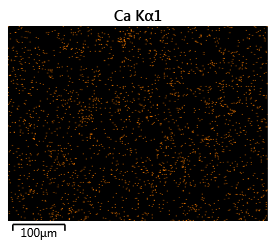

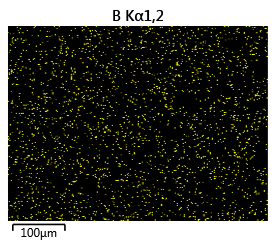

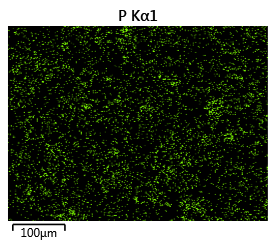

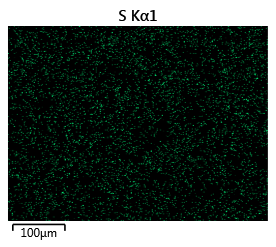

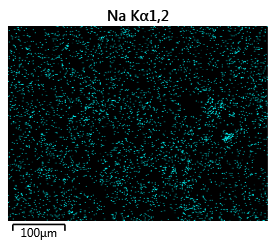

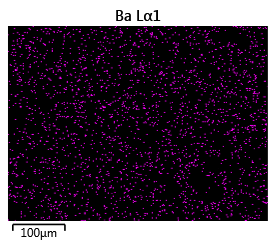


B)

Supplemental Figure 2.

Supplemental Figure 3.
